# Two-Capacitor Direct Interface Circuit for Resistive Sensor Measurements

**DOI:** 10.3390/s21041524

**Published:** 2021-02-22

**Authors:** José A. Hidalgo-López, Óscar Oballe-Peinado, Julián Castellanos-Ramos, José A. Sánchez-Durán

**Affiliations:** 1 Departamento de Electrónica, Universidad de Málaga, Andalucía Tech, Campus de Teatinos, 29071 Málaga, Spain; oballe@uma.es (Ó.O.-P.); jcramos@uma.es (J.C.-R.); jsd@uma.es (J.A.S.-D.); 2 Instituto de Investigación Biomédica de Málaga (IBIMA), 29010 Málaga, Spain

**Keywords:** direct interface circuits, interface sensor, resistive sensor, time-based measurement, calibration methods, uncertainty

## Abstract

Direct interface circuits (DICs) avoid the need for signal conditioning circuits and analog-to-digital converters (ADCs) to obtain digital measurements of resistive sensors using only a few passive elements. However, such simple hardware can lead to quantization errors when measuring small resistance values as well as high measurement times and uncertainties for high resistances. Different solutions to some of these problems have been presented in the literature over recent years, although the increased uncertainty in measurements at higher resistance values is a problem that has remained unaddressed. This article presents an economical hardware solution that only requires an extra capacitor to reduce this problem. The circuit is implemented with a field-programmable gate array (FPGA) as a programmable digital device. The new proposal significantly reduces the uncertainty in the time measurements. As a result, the high resistance errors decreased by up to 90%. The circuit requires three capacitor discharge cycles, as is needed in a classic DIC. Therefore, the time to estimate resistance increases slightly, between 2.7% and 4.6%.

## 1. Introduction

The study and development of sensors is currently one of the most important research areas in electronics [1]. These devices capture variations in physical quantities and use appropriate electronic circuits to convert these variations into electrical signals that can subsequently be processed. An efficient way to extract the information provided by these electrical signals is by using programmable digital devices (PDDs) [2] directly connected to the sensors through a few additional elements. Different PDDs, such as complex programmable logic devices (CPLDs) [3], FPGAs [4] and microcontrollers [5,6], can be used to pre-process this information in line with the needs of the application in which the sensor is used. These are known as smart sensors [7,8].

Signal conditioning circuits and Analog-to-Digital Converters (ADCs) are typically used as part of the interface between the sensor and the PDD [9,10], although it is also possible to use simpler and less-expensive alternatives known as Direct Interface Circuits (DICs). These circuits allow the sensors to be connected to the PDDs using just a few passive components (resistors or capacitors), and in some cases, simple active components such as transistors or triggers [11]. The absence of signal conditioning circuits and ADCs makes DICs highly suitable for use along with any low-cost PDDs. However, PDDs with ADCs could use them for other purposes.

The first DICs were presented simultaneously in References [12] and [13]. These seminal works have been used to develop designs for different types of sensors, including purely resistive [11,14,15,16], capacitively-coupled resistive sensors [17], capacitive [18,19,20], inductive [21,22,23], and even for some that support the connection of sensors of any of these types [24,25]. However, since resistive sensors are commonplace in electronics, DICs designed to read this magnitude are also the most used.

**Figure 1:**
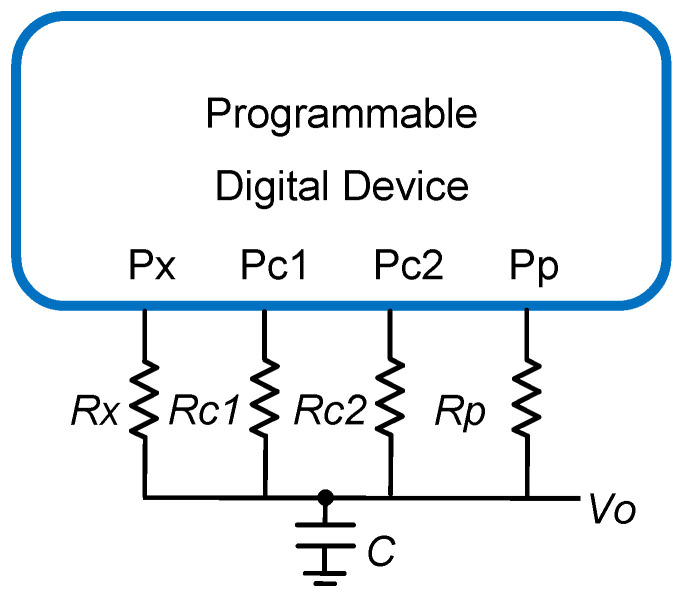
Classic direct interface circuit (DIC) for the readout of an *Rx* resistor based on the two-point calibration method (TPCM).

While the charge-transfer method may be used for a resistive DIC, as in Reference [16], this method requires complicated controls and has significant errors. In light of this, the simplest DICs for resistive sensors consist of a capacitor, *C*; the sensor, *Rx*; and two known-value calibration resistors, *Rc1* and *Rc2*, as passive components. Together with the PDD, these elements form the core of the sensor’s readout circuit, as shown in Figure 1. The PDD in this figure includes a counter (or timer, for microprocessors and microcontrollers) to determine, in PDD internal clock cycles, the duration of the different capacitor discharges through the calibration resistors and the sensor itself. 

The discharging states end when a reference voltage, *V_TH_* (the threshold voltage), is reached in the Pp pin in Figure 1, meaning the PDD detects a logic 0 in this pin. The Pp pin is configured as an input throughout the discharging state. Before these discharges, the capacitor is charged at the supply voltage, *V_DD_*, by the PDD, through Pp, Pc1, Pc2, and Px configured as a logic 1 output. As will be demonstrated below, operating with the times measured in the different discharges allows the sensor’s resistance value to be deduced. As the digital information generated by these circuits involves time measurements, the DICs actually perform a magnitude–time–digital conversion with minimal hardware, making them economical, easy to integrate, and with similar performance to using an ADC [10,26].

**Figure 2:**
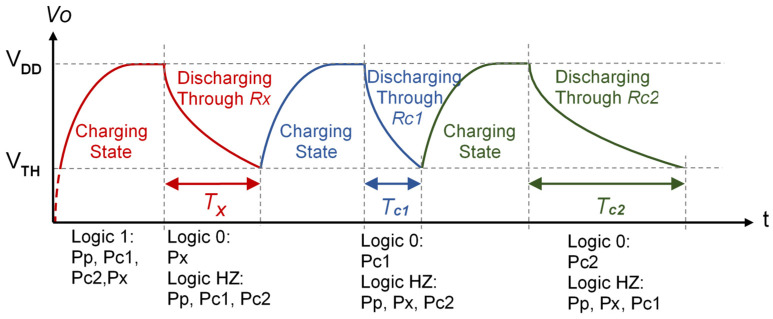
The charging and discharging stages to obtain the *T_X_*, *T_c1_*, and *T_c2_* times used in Equation (3) to estimate *Rx* using the TPCM with the DIC of Figure 1. For each of these stages, the state of the programmable digital device (PDD) pins is indicated under the time axis.

The method for analytically determining the sensor’s resistance value using the circuit in Figure 1, known as the Two-Point Calibration Method (TPCM), is simple and requires few calculation resources in the PDD. The four pins of the PDD in Figure 1 can be dynamically configured as outputs or inputs to use the TPCM. When configured as inputs, the PDD nodes remain in a high impedance state (HZ), from the circuit resistors’ point of view, thus preventing current from circulating through them. The whole measurement process is shown in Figure 2, and it consists of three capacitor charges made by placing logic 1 outputs in the Pp, Pc1, Pc2, and Px pins. 

This situation has been labeled as the “Charging State” in Figure 2. The *Rp* resistor in Figure 1 is added to improve the rejection of the power supply/noise interference [27], and its value should be minimal in order to speed up the charging processes. With *Rp*, the time measurements from the start of the discharge until *Vo(t)* = *V_TH_* (from now on, named as detection time) will be more independent of these noises. The capacitor is discharged through the different circuit resistors after each charging state by configuring the appropriate pin (Px, Pc1, or Pc2) as a logic 0 output. This procedure allows three times to be generated, corresponding to the discharge through each resistor, *T_x_*, *T_c1,_* and *T_c2_* in Figure 1. For each capacitor discharge, *Vo(t)* could change between the voltage for a logic 1, *V_DD_*, and the voltage for a logic 0, 0 V. Then

(1) $$Vo(t)=V_{DD}\cdot e^{\frac{-t}{(R+Ro)\cdot C}}$$

where *Ro* is the output resistor of the buffers of the PDD when configured as a logic 0 (*Ro* is considered a constant parameter in a first order approximation), and *R* is any resistor through which discharge is taking place. Thus, each of these three times has the form

(2) $$T=(R+Ro)\cdot C\cdot \ln\left(\frac{V_{DD}}{V_{TH}}\right)$$

From this expression, we can find *Rx* as

(3) $$R_{x}=\frac{T_{x}-T_{c1}}{T_{c2}-T_{c1}}(R_{c2}-R_{c1})+R_{c1}$$

In order for Equation (3) to improve the estimate of *Rx*, the values of *Rc1* and *Rc2* must be carefully selected to correct the errors that come from the fact that Equation (2) is an approximation to the true discharging time (a PDD output buffer is modeled as a constant value resistor). If the error term for *T* has a value proportional to *R*^2^ in the right-hand side of Equation (2), then (by the criteria set out in Reference [28]) *Rc1* and *Rc2* would be at 15% and 85% of the full-scale span of the resistances to be measured. This may not actually be the case in Equation (2), but regardless, this selection produces a suitable error profile, and for this reason, is commonly used in the literature.

Depending on the application of the sensor, in certain cases, the DIC in Figure 1 or the stages shown in Figure 2 must be modified. For example, the discharge time through *Rx* is short when measuring low resistance sensors, and there may be a significant error in determining its value because of the quantization in the clock cycles of the PDD. This can be solved by finishing the discharging states through a higher value resistor (any of the calibration resistors can be used for this purpose), as proposed in Reference [29], or by modifying the charging and discharging cycles [30]. The non-linearity of the resistors of the PDD buffers may also affect the estimates. In this case, the solution described in the literature is to use a calibration resistor in addition to those shown in Figure 1 [31]. In contrast, one of the problems for high resistance values is an excessive discharge time. This time can be reduced by making part of the discharge through a resistor that is smaller than *Rx* [32]. It is even possible to shorten the charging times (meaning the voltage stored in *C* at the end of each charging state may be different since the starting voltage in the charge is different) by proceeding, as set out in Reference [33]. In the case of remote resistive sensors, including several switches makes it possible to eliminate the influence of wire resistance in the measurements [14].

Uncertainty in the different time measurements is an important parameter when establishing the quality of the *Rx* estimate using the circuit in Figure 1. This uncertainty is mainly due to the electrical circuit noise that affects both the detection time and the voltage value actually stored in *C* at the end of each charging state. Noises are produced mostly by the switching activities of the PDD. However, they can also be due to the operation of other circuits that can be used together in the same electronic design. As commented above, including *Rp,* helps reduce the noise in the charging process of *C*. Thus, the main cause of uncertainty in the time measurements *T_x_*, *T_c1,_* and *T_c2_* is the noise that affects the detection time. 

Despite the importance of this parameter in the quality of measurements, no contributions have been reported to reduce this uncertainty. In fact, this uncertainty is the main cause of the maximum absolute errors that occur in a DIC [2]. This paper presents a modification of the circuit in Figure 1 that addresses this issue. The modification is simple and consists of adding a second capacitor to the circuit of Figure 1. Thus, the new design practically does not modify either the total time needed to estimate *Rx* or the circuit’s power consumption.

The structure of the paper is as follows. In Section 2, we present the operating principles of the proposed new DIC and includes a quantitative analysis of its operation. In Section 3, we show the Materials and Methods used when evaluating the new DIC, while in Section 4, we present the experimental results. Finally, the conclusions of this paper are presented in Section 5.

## 2. Operating Principle

If we define the uncertainty of measurement of a discharging time as the standard deviation that appears in multiple measurements, then the uncertainty in determining the detection time in a DIC, *u(T)*, is proportional to the value of the electrical noise present in the detection node and inversely proportional to the slope of the discharging curve in the detection time [2]:

(4) $$u(T)=\frac{k}{{\left|\frac{dVo(t)}{dt}\right|}_{t=T}}$$

where *k* is a constant related to the noise level in the circuit. Taking into account Equation (1), we can write:

(5) $$u(T)=\frac{k\cdot (R+Ro)\cdot C}{V_{TH}}$$

Therefore, once a value has been chosen for *C*, uncertainty in the discharge time measurement through a resistor increases as the resistance value increases. Similarly, as shown in Equation (3), *Rx* has a linear dependence on *T_x_*, meaning that the uncertainty in estimating *Rx* in a DIC, *u(Rx)_DIC_*, also increases with the value of *Rx* due to the law of propagation of uncertainty (except for low values of resistances where uncertainties due to quantization are important).

Equation (5) only shows two ways of decreasing *u(T)*: the reduction of *k* and/or *C*. However, in a real circuit, there will always be a minimum noise level that cannot be reduced, even with a careful design. As for *C*, a decrease in the capacitance may cause significant quantification errors when estimating lower resistance values, meaning that this option may have to be ruled out.

**Figure 3:**
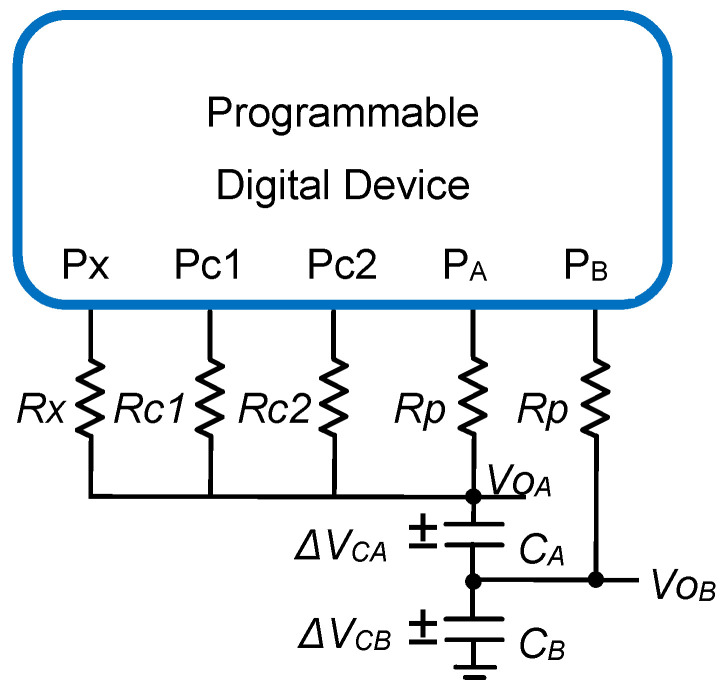
The two-capacitor direct interface circuit (TCDIC) proposed in this article. The circuit uses two capacitors, *C_A_* and *C_B_*, replacing *C* in Figure 1. The pins, P_A_ and P_B_, measure the times needed to estimate *Rx*.

This article proposes an alternative way to reduce *u(Rx)*. The basic idea is to modify the classic DIC in Figure 1 by replacing the single capacitor with a set of two capacitors, *C_A_* and *C_B_*, placed in series, as shown in Figure 3. This new circuit will be known as the two-capacitor DIC (TCDIC). With this modification, the TCDIC has two nodes (A and B) in which the voltages, *Vo_A_* and *Vo_B_*, can reach the value *V_TH_* at different moments of the same discharge cycle. These moments will be detected by pins P_A_ and P_B_. The circuit works properly if the values of *C_A_* and *C_B_* meet certain conditions for their capacities, as well as requiring a correct voltage at the end of every charging stage.

The TCDIC in Figure 3 consists of the same number of charging and discharging stages, as shown in Figure 2 for the classic DIC based on TPCM. The only difference is that two nodes now have to be charged and discharged. In the charging state of TCDIC, the P_A_ and P_B_ pins are configurated as logic 1 outputs, placing the voltage *V_DD_* in A and B. This actually performs the full discharge of *C_A_* and a charge of *C_B_*; however, for clarity, we will continue to call this time interval the charging state. These will be the initial conditions for the discharging states. The other resistors help to establish the voltage *V_DD_* in node A during the charging state, configuring pins Px, Pc1, and Pc2 as logic 1 outputs.

**Figure 4:**
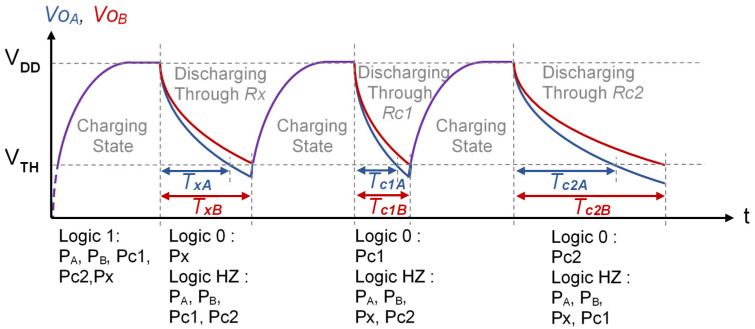
Steps necessary for the operation of the TCDIC shown in Figure 3. The time is measured in node A and another time is measured in node B in each discharge stage. Thus, we have *T_xA_*, *T_xB_*, *T_c1A_*, *T_c1B_*, *T_c2A_*, and *T_c2B_*, which will then be used to estimate *Rx*.

In the TCDIC discharging states, the P_A_ and P_B_ pins are configured as inputs (HZ), while Px, Pc1, and Pc2 are configured in the three different discharges, as in the DIC of Figure 1. This procedure produces six detection times (two in each discharging state) and their six associated time measurements, *T_xA_*, *T_xB_*, *T_c1A_*, *T_c1B_*, *T_c2A_*, and *T_c2B_*, which allow *Rx* to be estimated (the letters A or B indicate the node the time is measured in). The steps to obtain these measurements with the initial conditions mentioned are shown in Figure 4, which also indicates the pin configuration of the PDD in each state. As will be shown below, these six time measurements and the values of *Rc1* and *Rc2* can be used to estimate the value of *Rx* in a relatively similar way as to Equation (3).

### 2.1. Obtaining Rx Using the TCDIC

With Δ*V_CA_(t)* and Δ*V_CB_(t)* representing the difference in the electrical potential between the terminals of the capacitors *C_A_* and *C_B_* at each time instant, as shown in Figure 3, we can establish a system of two first-order differential equations when the discharge is initiated through any resistor, *R*, thus equalizing the current through the resistor, *i_R_*, with those that extract charge from *C_A_* and *C_B_*, *i_CA_* and *i_CB_*,

(6) $$\begin{array}{l} i_{R}=i_{CA}\Rightarrow \frac{\Delta V_{CA}+\Delta V_{CB}}{R+Ro}=-C_{A}\frac{d\Delta V_{CA}}{dt}\\ i_{R}=i_{CB}\Rightarrow \frac{\Delta V_{CA}+\Delta V_{CB}}{R+Ro}=-C_{B}\frac{d\Delta V_{CB}}{dt} \end{array}\}$$

For convenience, the solutions for this system of equations are written in the form:

(7) ΔVCA(t)=K1e−t(R+Ro)⋅Ceq+K2ΔVCB(t)=CACBΔVCA+K3

where *K_1_*, *K_2_*, and *K_3_* are constants that depend on the difference in electrical potential between the capacitor terminals, both at the start of the discharge stage, Δ*V_CA_(0)* and Δ*V_CB_(0)*, and also at the end if extended indefinitely, Δ*V_CA_(t→∞) + ΔV_CB_(t→∞) =* 0. *Ceq* is the equivalent capacitance of the two capacitors connected in series, *Ceq* = *C_A_*ǁ*C_B_*. With these initial conditions, two equations in Equation (7) can be written as:

(8) ΔVCA(t)=ΔVCA(0)+CBCA+CB⋅(e−t(R+Ro)⋅Ceq−1)⋅(ΔVCA(0)+ΔVCB(0))ΔVCB(t)=ΔVCB(0)+CACA+CB⋅(e−t(R+Ro)⋅Ceq−1)⋅(ΔVCA(0)+ΔVCB(0))

These expressions allow for finding the voltages in nodes A and B during the discharging states, *Vo_A_(t)* and *Vo_B_(t).*

(9) VoA(t)=ΔVCA(t)+ΔVCB(t)=(ΔVCA(0)+ΔVCB(0))⋅e−t(R+Ro)⋅CeqVoB(t)=ΔVCB(t)=ΔVCB(0)+CACA+CB⋅(e−t(R+Ro)⋅Ceq−1)⋅(ΔVCA(0)+ΔVCB(0))

As indicated in the previous section, establishing Δ*V_CA_(0)* = 0 and Δ*V_CB_(0)* = *V_DD_* during the Charging State will mean that these equations are transformed into:

(10) VoA(t)=VDD⋅e−t(R+Ro)⋅CeqVoB(t)=VDD⋅(CACA+CBe−t(R+Ro)⋅Ceq+CBCA+CB)

The detection times in nodes A, *T_A_*, and B, *T_B_*, therefore, occur in:

(11) $$\begin{array}{c} T_{A}=\left(R+Ro\right)\cdot Ceq\cdot \ln\left(\frac{V_{DD}}{V_{THA}}\right)\\ T_{B}=\left(R+Ro\right)\cdot Ceq\cdot \ln\left(\frac{V_{DD}\cdot C_{A}}{V_{THB}\cdot \left(C_{A}+C_{B}\right)-V_{DD}\cdot C_{B}}\right) \end{array}$$

with *V_THA_* and *V_THB_* as the threshold voltages in node A and B, respectively. Replacing *R* with *Rx*, *Rc1*, and *Rc2* in Equation (11) gives us the six detection times to estimate *Rx* using the TCDIC through the following expression:

(12) $$Rx=\frac{T_{xA}+T_{xB}-\left(T_{c1A}+T_{c1B}\right)}{T_{c2A}+T_{c2B}-\left(T_{c1A}+T_{c1B}\right)}\cdot \left(Rc2-Rc1\right)+Rc1$$

It is important to note that this expression is valid, even though *V_THA_* and *V_THB_* are different. However, in practice, this difference is small, and so we can take *V_TH_* = *V_THA_* ≈ *V_THB_*. It is easy to check, in Equation (11), that *T_B_* > *T_A_* (this is the situation shown in Figure 4).

Next, we will demonstrate that, if the time constants for discharge through the same resistor in the DIC and TCDIC are equal, then:

(a) Uncertainty in the estimate of *Rx* using a TCDIC and Equation (12), *u(Rx)_TCDIC_*, may be lower than the uncertainty of the estimate of *Rx* using a DIC and Equation (3), *u(Rx)_DIC_*. This is guaranteed if *C_A_* >> *C_B_*.
(b) If *C_A_* >> *C_B_*, the total time for estimating *Rx* is similar in both DICs.

For the DIC and TCDIC to have the same time constant discharging through the same resistance, it is necessary that *Ceq* = *C*. If this is verified, from Equation (2) and Equation (11):

(13) $$\begin{array}{c} T_{A}=T\\ T_{B}=\alpha \cdot T \end{array}$$

where *α* is:

(14) $$\alpha =\frac{\ln\left(\frac{V_{DD}\cdot C_{A}}{V_{TH}\cdot \left(C_{A}+C_{B}\right)-V_{DD}\cdot C_{B}}\right)}{\ln\left(\frac{V_{DD}}{V_{TH}}\right)}$$

As shown in Equation (14), the designer can, by selecting the *C_A_/C_B_* ratio, change *α* (the PDD used in the circuit sets the values of *V_DD_* and *V_TH_*). As the numerator argument is greater than the denominator argument, α is a positive number greater than one.

As for the uncertainties, *u(T_A_)* and *u(T_B_)* can be written using Equation (4) and Equation (10).

(15) $$\begin{array}{c} u\left(T_{A}\right)=\frac{k}{{\left|\frac{dVo_{A}(t)}{dt}\right|}_{t=T_{A}}}=\frac{k\cdot (R+Ro)\cdot Ceq}{V_{TH}}\\ u\left(T_{B}\right)=\frac{k}{{\left|\frac{dVo_{B}(t)}{dt}\right|}_{t=T_{B}}}=\frac{k\cdot (R+Ro)\cdot Ceq}{\left|V_{TH}-V_{DD}\frac{C_{B}}{C_{A}+C_{B}}\right|} \end{array}$$

To avoid that the denominator in the second equation is 0, it is necessary that:

(16) $$\frac{C_{A}}{C_{B}}>\frac{V_{DD}}{V_{TH}}-1$$

This is not a very demanding condition since, in practice, it is sufficient that *C_A_* is slightly higher than *C_B_*. Thus, from Equation (5) and Equation (15):

(17) $$\begin{array}{c} u\left(T_{A}\right)=u\left(T\right)\\ u\left(T_{B}\right)=\beta \cdot u\left(T\right) \end{array}$$

with *β* determined by:

(18) $$\beta =\frac{V_{TH}}{V_{TH}-V_{DD}\frac{C_{B}}{C_{A}+C_{B}}}$$

Functions *α* and *β* are used in Theorem 1 of Appendix A to show the relationship between *u(Rx)_DIC_* and *u(Rx)_TCDIC_*, Equations (A1) and (A2). If *C_A_*/*C_B_* verifies the condition in Equation (16), then the function *θ*, defined in Appendix A, is a positive real-valued function and is a decreasing function for *C_A_*/*C_B_*. Therefore, there is a minimum value of *C_A_*/*C_B_* from which *θ* < 1, and thus, also *u(Rx)_TCDIC_* < *u(Rx)_DIC_*. Determining this value in practice is a difficult task since *θ* also depends on *V_TH_* and *V_DD_* (values that the designer may not know). It is not possible to solve Equation (A2) for *C_A_*/*C_B_* in a closed form. However, it is more useful to know that the minimum value of *θ, θ_min_*, is given by:

(19) $$\theta_{min}={\theta |}_{C_{A}\gg C_{B}}={\frac{1+\beta^{2}}{{\left(1+\alpha \right)}^{2}}|}_{C_{A}\gg C_{B}}=\;\frac{1+1^{2}}{{\left(1+1\right)}^{2}}=\;\frac{1}{2}$$

Therefore, we can consider *C_A_* >> *C_B_* as the criterion for optimal design in the TCDIC, and if this condition is met:

(20) $$u{\left(Rx\right)}_{TCDIC}\approx \;\;\frac{1}{\sqrt{2}}u{\left(Rx\right)}_{DIC}\;□$$

To prove statement (b), it is sufficient to check that, if *C_A_* >> *C_B_*, then *α* → 1, and Equation (13) shows that the time measurements *T*, *T_A_*, and *T_B_* are very similar (although, clearly, *T_B_* will always be slightly higher than *T_A_*).

The result of Equation (21) is limited by the approximations used in Equations (2) and (11). Equation (21) will also have practical effects for resistances where the quantization uncertainty has little influence (typically medium and high resistances). However, it is precisely for high resistances where, when using a DIC, the uncertainty in the estimate of *Rx* is higher [2]. Therefore, the TCDIC improves the estimate precisely where this improvement is most needed.

### 2.2. Power Consumption of the TCDIC

Energy consumption in the TCDIC is very similar to that found for the DIC in Figure 1. To demonstrate this, we assumed that the discharges in the capacitors are completed in both Figure 2 and Figure 4, meaning that *Vo* = 0 V and *Vo_A_* = 0 V at the start of the charging states. In the case of the DIC in Figure 1, the power to be supplied to charge the capacitor, *E_DIC_*, is:

(21) $$E_{DIC}=C\cdot V_{DD}^{2}$$

In TCDIC, the power supplied to charge *C_B_*, *E_TCDIC_*(*C_B_*), is:

(22) $$E_{TCDIC}(C_{B})=C_{B}V_{DD}^{2}-C_{B}\cdot {\left(\frac{V_{DD}\cdot C_{B}}{C_{A}+C_{B}}\right)}^{2}$$

where the second term of the member on the right of Equation (22) is due to the fact that, when *Vo_A_* = 0, *Vo_B_* has a different value, which can be found via *t*→∞ in Equation (10). For this same reason, the energy supplied to *C_A_* during the charging state, *E_TCDIC_*(*C_A_*), is:

(23) $$E_{TCDIC}(C_{A})=C_{A}\cdot {\left(\frac{V_{DD}\cdot C_{B}}{C_{A}+C_{B}}\right)}^{2}$$

Adding the results of Equation (22) and Equation (23), the energy supplied in the TCDIC charging state, *E_TCDIC_*, can be found through some simple algebraic operations.

(24) $$E_{TCDIC}=E_{TCDIC}(C_{A})+E_{TCDIC}(C_{B})=C_{eq}V_{DD}^{2}\left(1+\frac{2C_{B}}{C_{A}+C_{B}}\right)$$

If the criterion for the optimal design is followed, then the result in Equation (24) is similar to that found in Equation (21), meaning that *E_DIC_* ≈ *E_TCDIC_*.

For all the above, the only true drawback of the TCDIC in Figure 3 compared to the DIC in Figure 1 is an increase in the hardware complexity, as it is necessary to add a second capacitor and use an additional PDD pin. However, the internal design in the PDD does not show any significant variation between the two circuits since, as in the case of the DIC, the TCDIC only requires a single counter to generate the six time measurements used in Equation (12). In contrast, Equation (12) is clearly more complex than Equation (3), even though it only involves two further additions and subtractions. There is also, obviously, the cost of storing six variables instead of the three used in the DIC.

Finally, the two capacitors in the TCDIC could also be used in a classic DIC based on the three-signal auto-calibration technique [34]. Similarly, the TCDIC may be compatible with other circuits designed to obtain different improvements in the characteristics of a resistive DIC, such as those shown in References [14,29,31,32,33], meaning that these circuits could also have benefits provided by the TCDIC.

## 3. Materials and Methods

To evaluate the performance of the TCDIC, a prototype of the circuit in Figure 3 was designed using a Spartan 6 FPGA (XC6SLX25-3FTG256) [35] from Xilinx (San Jose, CA, USA) running at 50 MHz as a PDD. The crystal clock oscillator used was the ASFL1 from Abracon Corporation (Spicewood, USA) with a frequency stability of ± 30 ppm with a 1 ps phase jitter RMS. Therefore, the uncertainty in the measurement of time caused by the clock oscillator is practically negligible. Any small change in the clock frequency will be offset by the calibration method. 

The FPGA was programmed in VHDL to perform the functions of the TCDIC’s PDD. This FPGA requires two different supply voltages; thus, a TPS79633 voltage regulator from Texas Instruments (Dallas, USA) was used for the 3.3 V supply voltage of the input/output blocks. This is the *V_DD_* voltage used in the description of TCDIC operation, meaning that the *V_TH_* is approximately 1.36 V. The FPGA core operates on a lower supply voltage of 1.2 V generated by a Texas Instrument TPS79912 voltage regulator to reduce the overall power consumption. 

The maximum current an output buffer of the FPGA can sink to maintain the digital signal integrity of the outputs is 24 mA. The prototype was manufactured with a FR-4 fiberglass reinforced epoxy-laminated substrate with four layers of conductive pathways. The internal layers were used for supply planes, and the external layers were used for the remaining signals.

In all cases, polypropylene film capacitors were used to compare the performance of the circuits in Figure 1 and Figure 3. A capacitor with a nominal value of 47 nF was selected for the circuit in Figure 1. This value of *C* was chosen so that the discharge times would not be excessively long and can be determined with a 14-bit counter, while also providing sufficient measurement accuracy. The same 47 nF capacitor was used for *C_B_* in Figure 3, while two different values were used for *C_A_* (560 and 820 nF) to compare the effects of the ratio *C_A_*/*C_B_* on the TCDIC performance.

To understand the values selected for *C_A_*, it is necessary to observe the values of function *θ* in our setup (*V_DD_* = 3.3 V, *V_TH_* = 1.36 V, and *C_B_* = 47 nF), as shown in Table 1.

From Equation (16), the minimum possible value for *C_A_* is 67 nF, and from this value there is a rapid drop in the values of *θ*, so that, for *C_A_* > 400 nF, *θ* is very close to its minimum value (in our setup *θ* = 1 for *C_A_* =141.3 nF). Thus, the choice of 560 nF and 820 nF for *C_A_* allows values of *θ* very close to its lower limit, and at the same time, the analysis of the performance of the circuit for different values of *C_A_*.

A total of three series of tests were carried out: one for the DIC and two for the TCDIC. A set of 21 resistors ranging from 128 to 7400 Ω were used in each series (the maximum discharge time for these resistors was 349.9 μs). This range coincides with that of a resistive tactile sensor used by the authors. It was manufactured with a sheet of piezoresistive material by the company CIDETEC (San Sebastián, Spain) [36]. 

Two calibration resistors were used for all tests, at approximately 15% and 85% of the full resistance range, *Rc1* = 1100 Ω and *Rc2* = 4600 Ω. All the resistors were measured using an Agilent 34401A digital multimeter. A total of 500 measurement cycles were performed for each of the 21 resistors. These measurements were used to evaluate the errors and uncertainties for each of the two possible capacitor combinations.

To improve detection of the trigger event in P_A_ and P_B_, the circuits proposed in Reference [37] were implemented in the FPGA for both pins. These modules avoid the need to use Schmitt triggers or similar circuitry if there are fluctuations in the buffer voltage around *V_TH_*. 

## 4. Experimental Results

Next, we show and discuss the results of three series of tests carried out with the set up indicated in the previous section.

As a consequence of the *C_A_* and *C_B_* values selected, the capacitance of the equivalent discharge capacitor in node A, *Ceq*, is slightly lower than 47 nF, since *C_B_* was chosen with this capacitance in all cases. However, as *C_A_* >> *C_B_*, *Ceq* is very close to *C* (*Ceq* = 43.36 nF for *C_A_* = 560 nF and *Ceq* = 44.45 nF for *C_B_* = 820 nF). Thus, uncertainty in the measurement of times in the Pp pins of the DIC and P_A_ of the TCDIC should be very similar for the same resistors in the three series of tests. 

**Figure 5:**
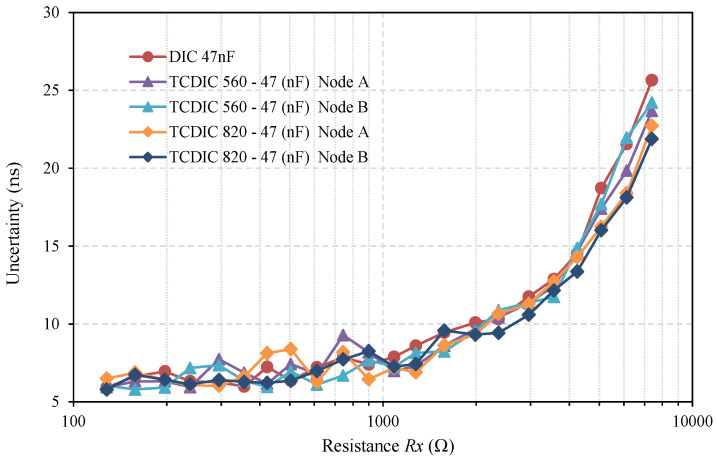
Uncertainty in the measurement of the discharge times in the node corresponding to the DIC’s Pp pin, *T*, and in nodes A and B of the TCDIC, *T_A_* and *T_B_*. The X-axis, in log10 scale, shows the resistors used.

As the criterion for the optimal design is met, Equation (15) indicates that these uncertainties must also be very similar to those obtained through the TCDIC pin P_B_. This can be seen in Figure 5, which shows the uncertainty curves (expressed in ns) when measuring the discharging times through each of the series resistors, and for the three sets of tests carried out. The curve labeled “DIC 47 nF” shows uncertainty in the discharge time measurement in the DIC in Figure 1, while the rest of the curves show the uncertainties of the time measurements for the different TCDIC configurations.

The results of the uncertainties in both nodes A and B are shown for each of the configurations of the TCDIC. As can be seen in Figure 5, all uncertainties had very similar values and showed the typical profile of increasing with the value of the resistor to be measured [2]. In terms of the circuit operation, the results in Figure 5 first show a confirmation of the equality of the uncertainties for the measurements in the DIC and the TCDIC, as Equation (17) predicts whether *C_A_* >> *C_B_* is met; secondly, the results ensure that having two capacitors in the circuit together with two measurement pins does not deteriorate the quality of the measurements.

As *C_A_* >> *C_B_*, in both configurations of TCDIC, *α* → 1 and following Equation (13), *T*, *T_A,_* and *T_B_* must be very similar. For example, for *Rx* = 3570 Ω, a resistance in the middle of the range, the mean of the 500 measurements in the DIC is *T_x_* = 142.42 µs. For *C_A_* = 560 nF, the means in the TCDIC are *T_A_* = 132.83 µs and *T_B_* = 152.93 µs. For *C_A_* = 820 nF, the means are *T_A_* = 135.12 µs and *T_B_* = 147.4 µs. The small difference between *T* and *T_A_* is due to the fact that the *Ceq* is slightly different from *C* for both configurations. However, *T_B_* differs from *T_A_* because *α* is not exactly 1. The fact that *T_B_* is somewhat greater than *T* also implies that the time required to estimate *Rx* is slightly longer in the TCDIC compared with in the DIC. Finally, as predicted by Equations (13) and (14), an increase in *C_A_* (with a constant *C_B_*) implies a decrease in *T_B_*.

In our case, all the arithmetic operations necessary to obtain *Rx*, from Equations (3) and (12), were performed in the FPGA during the charging and discharging states. Therefore, the time required to estimate *Rx* and transmit its value is only the sum of the three charging and discharging times (the ability of an FPGA to perform parallel computing avoids the need to stop the PDD operation during the charging and discharging states, such as in microprocessor or microcontroller implementations). As the charging time that was selected to guarantee a correct charging of the capacitors, in all cases, was 163.84 µs, the total times to estimate *Rx* = 3570 Ω, in the DIC, *TT(Rx) _DIC_*, and in the TCDIC, *TT(Rx) _TCDIC_*, are (with *C_A_* = 560 nF):

(25) $$\begin{array}{l} TT{\left(Rx\right)}_{DIC}=T_{x}+T_{c1}+T_{c2}+491.52\;\mu s\\ TT{\left(Rx\right)}_{TCDIC}=T_{xB}+T_{c1B}+T_{c2B}+491.52\;\mu s \end{array}$$

The maximum values for these times, *TT_max,DIC_* and *TT_max,TCDIC_*, are for the highest resistance in the range. Thus, it has been obtained for the DIC *TT_max,DIC_* = 1003.4 µs, and for the TCDIC, with *C_A_* = 560 nF, *TT_max,TCDIC_* = 1051.8 µs. If *C_A_* = 820 nF, *TT_max,TCDIC_* is 1031.7 µs. With these results, in our setup, the TCDIC is 4.6% slower than the DIC, if *C_A_* = 560 nF, and 2.7%, if *C_A_* = 820 nF. With these times, the equivalent sampling frequency of *Rx* is approximately 1 kHz for TCDIC and DIC.

**Figure 6:**
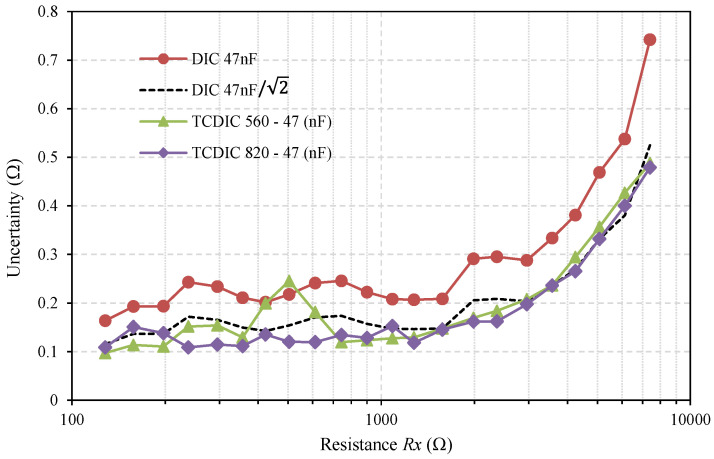
Uncertainty, expressed in Ω, in estimating *Rx* (calculated from 500 estimates) using a classic TPCM DIC, with *C* = 47 nF (red curve) and the TCDIC in two different configurations: *C_A_* = 560 nF-*C_B_* = 47 nF (green curve), and *C_A_* = 820 nF-*C_B_* = 47 nF (violet curve). The black curve shows, for comparison with the theoretical results, the uncertainty in the DIC divided by $\sqrt{2}$.

Figure 5 confirms the equality in the time measurements uncertainty, and as the time measurements are very similar, we expected that *u(Rx)_TCDIC_* < *u(Rx)_DIC_* will be confirmed. This improvement can be seen in Figure 6. This figure shows the uncertainty obtained in estimating *Rx* (expressed in Ω) from the 500 estimates made for each of the three series of tests. The uncertainty curves of this figure more clearly illustrate the benefits of the TCDIC over the TPCM-based DIC. 

The shapes of the curves are very similar to those shown in Figure 5; however, here, the differences for each DIC are clearer, showing reductions in most cases. The black curve shows the *u(Rx)_DIC_*/$\sqrt{2}$ function. This is the curve that one would expect to find in the TCDIC considering that *C_A_* >> *C_B_* and *Ceq* ≈ *C* (*u(Rx)_TCDIC_* ≈ *u(Rx)_DIC_*/$\sqrt{2}$). For high resistance values, the results are in complete agreement with the theoretical predictions.

.

**Figure 7:**
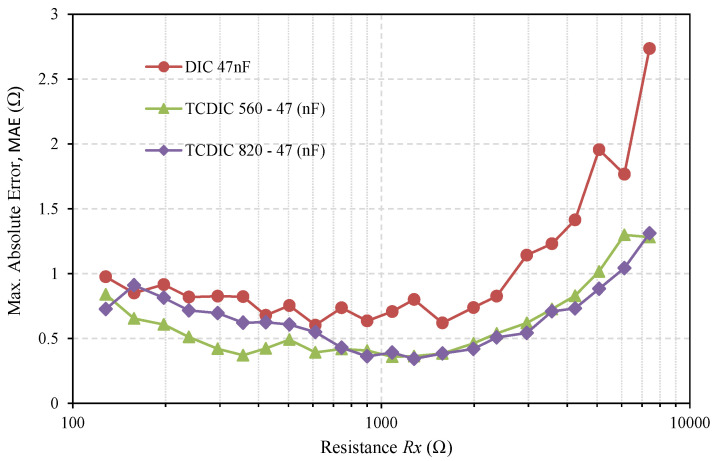
The maximum absolute error in estimating *Rx* using a classic TPCM DIC, with *C* = 47 nF (red curve), and the same two TCDIC configurations as shown in Figure 5 and Figure 6.

The reduction in *u(Rx)_TCDIC_* must be reflected in a better estimate of *Rx*. This can be seen in Figure 7, which shows the Maximum Absolute Error in estimating *Rx* (in Ω), *MAE(Rx)*, defined as:

(26) $$MAE(Rx)=Max\left(\left|Estimated\;Value{\left(Rx\right)}_{i}-Actual\;Value(Rx)\right|\right)\;i\in \left\{1,2,\ldots ,500\right\}$$

where *i* indicates the number of the estimate (MAE(Rx) is the “worst” case of the 500 measurements). As expected, Figure 7 shows that higher values of *MAE(Rx)* occur when estimating higher resistance values. For these resistances, *MAE(Rx)* is always greater using the DIC in Figure 1 than in any of the TCDIC configurations. The decrease in *MAE(Rx)* is due not only to the reduction in *u(T_x_)*, but also to the reductions in *u(T_c1_)* and *u(T_c2_)*. Figure 7 also confirms that, for the lower resistances, the increasing quantization errors and errors due to approximations in Equations (2) and (11) make the situation more confusing. However, the errors in the DIC are greater than in the TCDIC in practically all cases.

At first glance, Figure 7 also shows that there is not actually any significant difference between using one configuration with *C_A_* = 560 nF and another with *C_A_* = 820 nF. To confirm this, and as a figure of overall merit, Table 2 shows the sum of the *MAE(Rx)* of all resistors for each of the three curves in Figure 7. As this table shows, the sum of the errors is very similar for *C_A_* = 820 nF and *C_A_* = 560 nF, as might be expected when the two configurations have similar values of *θ*.

However, Equation (24) shows that for *C_A_* = 560 nF and *C_B_* = 47 nF, the power consumption increased by 6.5%. For *C_A_* = 820 nF and *C_B_* = 47 nF, the increase was 4.8%. Therefore, there is a trade-off between *TT(Rx)_TCDIC_* and the power consumption; thus, the most suitable setting depends on the application. 

**Figure 8:**
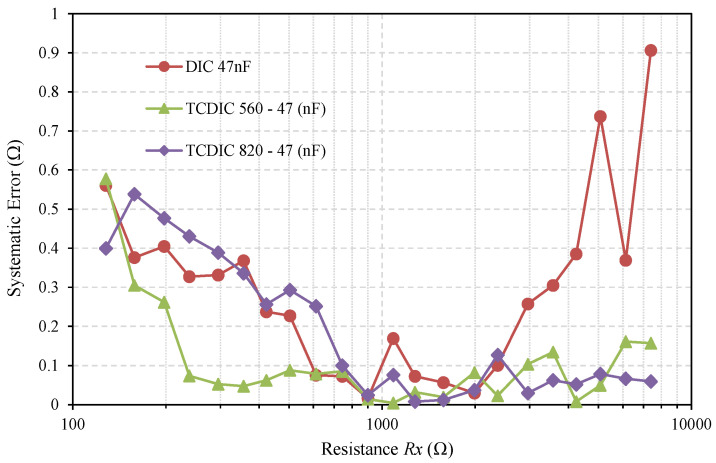
The systematic error in estimating *Rx* (calculated from 500 estimates) using a classic DIC, with *C* = 47 nF (red curve) and the same two TCDIC configurations as shown in Figure 6 and Figure 7.

Finally, in Figure 8, we compare the systematic error in estimating *Rx* through the two interface circuits. This systematic error is defined as the difference between the average of the 500 estimates made for *Rx* in each test with the actual resistance value. Once again, we see similar behavior to that shown in Figure 6 and Figure 7: the TCDIC has a clearly lower systematic error than the DIC for high resistances. The reduction in these cases was higher than 80% for the configuration with *C_A_* = 560 nF and higher than 90% for *C_A_* = 820 nF. As expected, the systematic errors for TCDIC and DIC were very similar due to the quantization errors for low resistance measurements.

Based on these results, TCDIC is a circuit that is particularly suitable for measuring medium-to-high resistances, as happens in tactile sensors when low pressures are applied. This situation is typical in grip tasks, and thus, in future works, we will include the TCDIC as a measurement circuit for this type of sensor.

## 5. Conclusions

Direct interface circuits (DICs) for the conversion of a sensor’s resistance value to digital information provide a simple and low-cost solution that is valid for virtually any programmable digital device. The initial versions of DICs had speed problems when estimating high resistance values and quantization errors for low resistance values. However, these have been minimized through different contributions from the literature. A further limitation that had not been addressed previously is that the uncertainty when measuring the time necessary to estimate a resistance value in a DIC is proportional to this value. 

The result is high uncertainty, and as a consequence, high error, whenever the resistance values are also high. This article proposes a simple solution for reducing the uncertainties, based on including a second capacitor and a measurement node. The solution presented, named the two-capacitor DIC (TCDIC), involves a slight increase in the DIC hardware, time, and power consumption required to estimate the resistance value. Six time measurements are needed instead of the three required by a two-point calibration method on a conventional DIC; however, the number of discharge stages does not increase from that used by the DIC. 

As a consequence, there is no significant increase in the power consumption or time needed for an estimate. The proposed measurement method and architecture for the TCDIC can be incorporated in the solutions described in the literature for reducing the measurement times or errors due to the quantization effects. Finally, design rules were proposed to maximize the TCDIC performance. As a proof of concept, the TCDIC circuit was implemented using an FPGA. For the highest resistances in this circuit, the errors were reduced by over 80%–90%. In contrast, the time needed for an estimate increased by only 2.7% to 4.6%, and the power consumption increased by 4.8% to 6.5%.

## Figures and Tables

**Table 1:** Function *θ* evaluated as a function of *C_A_* with *V_DD_* = 3.3 V, *V_TH_* = 1.36 V, and *C_B_* = 47 nF.

*C_A_* (nF)	Function *θ*
70	50.53
75	11.50
100	1.98
141.3	1.00
200	0.74
300	0.62
400	0.57
600	0.54
800	0.53
1000	0.52

**Table 2:** The table shows the sum of the Maximum Absolute Errors for each capacitor configuration.

*Capacitors Configuration*	$\sum_{Rx}MAE(Rx)$(Ω)
DIC *C* = 47 nF	22.6
TCDIC *C_A_* = 560 nF, *C_B_* = 47 nF	13.4
TCDIC *C_A_* = 820 nF, *C_B_* = 47 nF	14.3

## Data Availability

The processing codes and data segments can be obtained by contacting the corresponding author.

## Appendix A

**Theorem** **1.**
*Uncertainties in a classic DIC, u(Rx)_DIC_, and in the TCDIC, u(Rx)_TCDIC_, are related by:*

(A1) $$u^{2}{\left(Rx\right)}_{TCDIC}=\theta \cdot u^{2}{\left(Rx\right)}_{DIC}$$

*where function θ is defined as*

(A2) $$\theta =\theta \;(V_{DD},V_{TH},\frac{C_{A}}{C_{B}})=\frac{1+\beta^{2}}{{\left(1+\alpha \right)}^{2}}$$

**Proof** **of** **Theorem** **1**. If we consider that *u(Rx)_DIC_* and *u(Rx)_TCDIC_* only depend on the time measurement uncertainties and that these uncertainties are uncorrelated (charging states ensure this), we can write, for *u(Rx)_DIC_*

(A3) $$\begin{array}{l} u^{2}{\left(Rx\right)}_{DIC}={\left(\frac{\partial Rx}{\partial T_{x}}\right)}^{2}u^{2}\left(T_{x}\right)+{\left(\frac{\partial Rx}{\partial T_{c1}}\right)}^{2}u^{2}\left(T_{c1}\right)+{\left(\frac{\partial Rx}{\partial T_{c2}}\right)}^{2}u^{2}\left(T_{c2}\right)\\ ={\left(\frac{Rc2-Rc1}{T_{c2}-T_{c1}}\right)}^{2}\left(u^{2}\left(T_{x}\right)+{\left(\frac{T_{x}-T_{c2}}{T_{c2}-T_{c1}}\right)}^{2}u^{2}\left(T_{c1}\right)+{\left(\frac{T_{x}-T_{c1}}{T_{c2}-T_{c1}}\right)}^{2}u^{2}\left(T_{c2}\right)\right)\\ ={\left(\frac{Rc2-Rc1}{T_{c2}-T_{c1}}\right)}^{2}\left(u^{2}\left(T_{x}\right)+{\left(\frac{Rx-Rc2}{Rc2-Rc1}\right)}^{2}u^{2}\left(T_{c1}\right)+{\left(\frac{Rx-Rc1}{Rc2-Rc1}\right)}^{2}u^{2}\left(T_{c2}\right)\right) \end{array}$$

and, for *u(Rx)*_*TCDIC,*_

(A4) $$\begin{array}{c} u^{2}{\left(Rx\right)}_{TCDIC}={\left(\frac{\partial Rx}{\partial T_{xA}}\right)}^{2}u^{2}\left(T_{xA}\right)+{\left(\frac{\partial Rx}{\partial T_{xB}}\right)}^{2}u^{2}\left(T_{xB}\right)+{\left(\frac{\partial Rx}{\partial T_{c1A}}\right)}^{2}u^{2}\left(T_{c1A}\right)\\ +{\left(\frac{\partial Rx}{\partial T_{c1B}}\right)}^{2}u^{2}\left(T_{c1B}\right)+{\left(\frac{\partial Rx}{\partial T_{c2A}}\right)}^{2}u^{2}\left(T_{c2A}\right)+{\left(\frac{\partial Rx}{\partial T_{c2B}}\right)}^{2}u^{2}\left(T_{c2B}\right)=\\ {\left(\frac{Rc2-Rc1}{T_{c2A}+T_{c2B}-T_{c1A}-T_{c1B}}\right)}^{2}\times \\ \left(\begin{array}{l} u^{2}\left(T_{xA}\right)+{\left(\frac{Rx-Rc2}{Rc2-Rc1}\right)}^{2}u^{2}\left(T_{c1A}\right)+{\left(\frac{Rx-Rc1}{Rc2-Rc1}\right)}^{2}u^{2}\left(T_{c2A}\right)+\\ +u^{2}\left(T_{xB}\right)+{\left(\frac{Rx-Rc2}{Rc2-Rc1}\right)}^{2}u^{2}\left(T_{c1B}\right)+{\left(\frac{Rx-Rc1}{Rc2-Rc1}\right)}^{2}u^{2}\left(T_{c2B}\right) \end{array}\right) \end{array}$$

Using Equations (13), (17), and (A3), Equation (A4) can be written

(A5) $$\begin{array}{c} u^{2}{\left(Rx\right)}_{TCDIC}=\frac{1+\beta^{2}}{{\left(1+\alpha \right)}^{2}}{\left(\frac{Rc2-Rc1}{T_{c2}-T_{c1}}\right)}^{2}\times \\ \left(u^{2}\left(T_{x}\right)+{\left(\frac{Rx-Rc2}{Rc2-Rc1}\right)}^{2}u^{2}\left(T_{c1}\right)+{\left(\frac{Rx-Rc1}{Rc2-Rc1}\right)}^{2}u^{2}\left(T_{c2}\right)\right)=\\ =\frac{1+\beta^{2}}{{\left(1+\alpha \right)}^{2}}u^{2}{\left(Rx\right)}_{DIC}=\theta \cdot u^{2}{\left(Rx\right)}_{DIC}\;□ \end{array}$$

 □
